# Early Influence of Affective Context on Emotion Perception: EPN or Early-N400?

**DOI:** 10.3389/fnins.2018.00708

**Published:** 2018-10-16

**Authors:** Nerea Aldunate, Vladimir López, Conrado A. Bosman

**Affiliations:** ^1^Escuela de Psicología, Pontificia Universidad Católica de Chile, Santiago, Chile; ^2^Cognitive and Systems Neuroscience Group, Swammerdam Institute, Center for Neuroscience, University of Amsterdam, Amsterdam, Netherlands; ^3^Research Priority Program Brain and Cognition, University of Amsterdam, Amsterdam, Netherlands

**Keywords:** EPN, N400, attentional resources, early emotional processing, context, emotional perception, congruence, saliency

Recent studies have shown that affective context influences the processing of emotional images at early stages. Yet, the mechanisms underlying the emotional modulation of early sensory processing have not been totally elucidated. Emotional information plays a relevant role modulating several cognitive processes such as perception (Vuilleumier et al., 2004), attention (Vuilleumier, 2005; Yiend, 2010), memory (Kensinger, 2009), semantic integration (Egidi and Nusbaum, 2012), and social cognition (Forgas, 2001; Gross, 2002; Batty and Taylor, 2003). Emotions are supported by dynamic interactions among neuronal networks, linking emotional processing centers (e.g., amygdala, hippocampus), with noticeable connections to early sensory areas (Pessoa, 2008). These influences have been characterized through the modulation of two different Event Related Potentials (ERPs): (a) the Early Posterior Negativity (EPN) (Hietanen and Astikainen, 2013), related with the amount of attentional resources to facilitate the early selective perceptual processing of emotional stimuli (Junghöfer et al., 2001; Schupp et al., 2003); and (b) the early-N400 (Diéguez-Risco et al., 2015; Dozolme et al., 2015), classically related with the contextual influence on the effort to integrate incoming information in semantic processing (Kutas and Federmeier, 2011). Here, we discuss that the variations in latency, topography, and amplitude reported in these previous studies as an early-N400 component, fit better with those modulations observed in the EPN component, suggesting that affective context influences the allocation of attentional resources for emotional stimuli processing at early stages of perception.

## EPN and their role in selective attention during perceptual processing of emotional stimuli

EPN is a negative-going deflection over temporo-occipital sites, peaking between 250 and 350 ms after visual stimulus presentation. It has been considered as a marker of the earliest processing of selective emotional perception in relation to the amount of attentional resources (Junghöfer et al., 2001; Schupp et al., 2004a,b). The amplitude of the EPN correlates with emotional arousal (Junghöfer et al., 2001; Schupp et al., 2004a,b) and with emotional content of pictures (Low et al., 2005). The emotional information associated to stimulus content influences its salience (Öhman et al., 2000). Critically, the amount of allocated attentional resources correlates with the stimulus emotional salience (Ohman et al., 2001; Vuilleumier, 2005). This relationship indicates an attentional flexibility to modulate perceptual processing as a function of stimulus emotional content (Tipples and Sharma, 2000). Therefore, EPN amplitude reflects the amount of attentional resources allocated to salient stimuli, and the interaction between emotional information and attentional direction (Schupp et al., 2004a,b, 2006a).

Several studies have addressed the relationship between attentional resources and emotional salience of stimuli, observing EPN modulations (Schupp et al., 2003, 2006a; Kissler et al., 2009). Using rapid serial visual presentation paradigms (RSVP), it has been shown smaller EPN amplitudes in those stimuli that were preceded by emotional pictures (Flaisch et al., 2008a,b), suggesting that stimulus emotional relevance guides attentional resources assigned to perceptual processing (Lang et al., 1997; Codispoti et al., 2006; Schupp et al., 2007b). These findings have been interpreted as an emotional modulation of attentional salience and engagement in the first picture, interfering with subsequent emotional pictures processing (McHugo et al., 2013). This has been considered an “emotional blink,” quantified through EPN amplitude (Schupp et al., 2003). However, this effect is unspecific regarding emotional valence of the first picture, and the emotional content of the subsequent one (Most et al., 2007). Thus, aside from the emotional modulation of attentional processing, EPN amplitude in RSVP paradigms reflects the attentional engagement and competition between pictures (Shapiro et al., 1997; Potter et al., 2002). Its modulation can be interpreted as an early marker of attentional facilitation for the eliciting stimulus, with the subsequent depletion of attentional resources for competing one.

## Affective context modifies the allocation of attentional resources for emotional stimuli processing at early stages of perception

Recently, different studies have explored the influence of emotional context during emotional faces processing, showing that emotional context modulates early ERP amplitudes elicited by faces with emotional expressions (Hietanen and Astikainen, 2013; Diéguez-Risco et al., 2015; Dozolme et al., 2015). From these studies, Dozolme et al. (2015) and Diéguez-Risco et al. (2015) have interpreted these amplitude changes as early-N400 modulations. However, Hietanen and Astikainen (2013) interpret a similar negativity as an EPN modulation. Dozolme et al. (2015) recorded ERPs during the presentation of five emotional faces (sad, angry, fear, happy, neutral), that could be emotionally congruent or incongruent with a previous exposed situational sentence. After the presentation of emotional faces, they observed an occipital negative-going deflection between 200 and 340 ms, with larger amplitudes for the congruent condition. They interpreted this result as an early-N400. Nevertheless, they recognize that both, posterior localization and reversed congruence effects are inconsistent with a classical N400 component, finally suggesting that more research is needed to understand their results (Dozolme et al., 2015). In a similar study, Diéguez-Risco et al. (2015) presented two emotional faces (angry vs happy), which could be congruent or incongruent with a previously depicted situational sentence. They observed a posterior negative deflection between 250-350 ms, also interpreted as N400. This early negativity exhibited enhanced amplitude for congruent condition, without statistically significant congruence effects. Altogether, Dozolme et al. (2015) and Diéguez-Risco et al. (2015) interpreted these putative N400 modulations as a reflection of emotional contextual influence in the process of information integration. The fact that the negative component observed in these studies was labeled as an N400 might be related with the long literature reporting this component in experimental designs that contrast congruency between context and critical stimuli (Kutas and Federmeier, 2011).

Yet, the interpretation of these early negativities as N400 does not provide a clear explanation on how the affective context influences the processing of emotional information. The reason of this is twofold. First, considering their hedonistic condition (pleasant vs. unpleasant), the number of stimuli differs between the experiments. The effects of emotional content in congruent conditions obtained by Dozolme et al. (2015) and Diéguez-Risco et al. (2015) can be explained by the lack of counterbalanced distribution of stimuli regarding their affective valence (positive/pleasant vs. negative/unpleasant). Dozolme et al. (2015) included three emotions with negative valence (sad, fear, angry), but only one emotion with positive valence (happy). Conversely, positive and negative affective valences (happy and angry faces) were equally distributed in Diéguez-Risco et al. (2015). Additionally, Diéguez-Risco et al. (2015) suggested that the number of introduced emotions in the task can account for amplitude differences in early components. According to them, changes in the component amplitude imply different cognitive efforts. Specifically, the use of five emotions in Dozolme et al. (2015) increases the cognitive effort to solve the task, as compared with only two emotions (Diéguez-Risco et al., 2015).

The second reason is that this emotionally modulated negative component may correspond to an EPN. Specifically, there are major discrepancies between the observed 200–340 ms negativity, with a classical N400 in latency, topography and amplitude modulation. Classically, N400 is a negative-going deflection with enhanced amplitudes during the presentation of incongruent stimuli. This ERP component has been functionally related to the degree of effort during meaning comprehension and expectancy (Kutas and Federmeier, 2011). Also, N400 is modulated by faces, pictures, or gestures (Nigam et al., 1992; Debruille et al., 1996; Cornejo et al., 2009), among others stimuli. Different studies have shown that N400 amplitude is affected by different contextual factors like discourse (Otten and Van Berkum, 2007), music (Koelsch et al., 2004), world knowledge (Hagoort et al., 2004), prosodic cues (Steinhauer et al., 1999), or pitch accent (Wang et al., 2011). Altogether, these findings suggest that N400 signs the influence of context on expectancy and meaning comprehension.

The maximum peak of the negative component found in the studies of Diéguez-Risco et al. (2015) and Dozolme et al. (2015) was observed around 270 ms. Classically, N400 peaks around 400 ms, with a range between 300 and 500 ms (Kutas and Federmeier, 2011), whereas the EPN peak is usually observed between 250 and 350 ms (Schupp et al., 2004b). Additionally, this early negative deflection is located at posterior sites, whereas N400 is mostly located at fronto-parietal sites, rather anterior compared with the location of the observed negativity. Furthermore, the observed congruence effect in these studies was reverted compared with what is usually expected on an N400 congruence effect, obscuring the functional interpretation. Considering the accepted interpretation of the classically reported N400 component, the results of Diéguez-Risco et al. (2015) and Dozolme et al. (2015) would imply that congruent affective contexts involve a greater effort to integrate emotional stimuli. On the contrary, if these early negativities are considered as EPN, their amplitude modulation would indicate that the affective congruent context also influences the processing of emotional stimulus at very early stages, through an attentional facilitation mediated by its emotional content.

A third study provides further support to the hypothesis that affective context influences early stages of selective attentional perception (EPN). In an emotional classification task, Hietanen and Astikainen (2013) explored the influence of contextual scenes on emotional face perception. They recorded brain activity for happy and sad faces preceded by congruent and incongruent scenes. Participants were instructed to attend to faces and classified them as happy or sad. The authors reported a modulated EPN component between 250 and 350 ms at posterior regions, with larger amplitude for happy faces, in alignment with Diéguez-Risco et al. (2015) reports, among others (Schupp et al., 2006b; Flaisch et al., 2008a; Franken et al., 2008). They also observed a congruence effect for happy faces. EPN amplitude was larger when faces were preceded by congruent contexts. Importantly, the topography and latency of the elicited negative component were similar to those observed in the Dozolme et al. (2015) and Diéguez-Risco et al. (2015) studies. Considering that EPN reflects the amount of attentional resources in early selective processing of emotions, enhanced EPN amplitudes for congruent conditions might reflect more allocation of attentional resources during emotional stimulus processing. The enhanced effect observed for pleasant emotional information has been previously reported, suggesting a natural bias that facilitate the processing of such emotional stimuli (Herbert et al., 2008). Accordingly, new stimulation paradigms, including parameterization of hedonic information during context, together with the quantification of EPN amplitude changes could help understand the role of emotional information during early attentional processing.

In conclusion, here we yielded a unitary interpretation of the observations of affective contextual influence on emotional stimuli processing and its ERPs correlates. Our interpretation assumes that changes in EPN amplitude reflect both, stimulus emotional significance and the amount of attentional resources on early processing (Schupp et al., 2007a; Dunning et al., 2011; Weinberg et al., 2013). Consequently, we suggest that the observed EPN amplitude differences for the same type of stimulus, but under different emotional contexts, imply that the emotional significance might be derived not only from the stimulus in itself, but also from the affective context. The fact that effect of affective context can be observed at such early latencies, may suggest that the brain can use these modulatory signals to improve the efficiency of neural representations, and optimize stimulus inference, in line with novel theories of efficient and predictive coding (Vinck and Bosman, 2016).

## Author contributions

NA Conception, analysis and interpretation of the literature, she has participated in drafting of the manuscript and critical revision of the manuscript for important intellectual content, final approval of the version to be submitted. VL Conception drafting of the manuscript and critical revision of the manuscript for important intellectual content; final approval of the version to be submitted. CB has participated in drafting of the manuscript and critical revision of the manuscript for important intellectual content; final approval of the version to be submitted.

### Conflict of interest statement

The authors declare that the research was conducted in the absence of any commercial or financial relationships that could be construed as a potential conflict of interest.

## References

[B1] Batty M. Taylor M. J. (2003). Early processing of the six basic facial emotional expressions. Cogn. Brain Res. 17, 613–620. 10.1016/S0926-6410(03)00174-5 14561449

[B2] Codispoti M. Ferrari V. Junghöfer M. Schupp H. T. (2006). The categorization of natural scenes: brain attention networks revealed by dense sensor ERPs. Neuroimage 32, 583–591. 10.1016/j.neuroimage.2006.04.180 16750397

[B3] Cornejo C. Simonetti F. Ibáñez A. Aldunate N. Ceric F. López V. (2009). Gestures and metaphorical comprehension: electrophysiological evidence of the influence of gestures on metaphorical processing. Brain Cogn. 70, 42–52. 10.1016/j.bandc.2008.12.005 19200632

[B4] Debruille J. B. Pineda J. Renault B. (1996). N400-like potentials elicited by faces and knowledge inhibition. Cogn. Brain Res. 4, 133–144. 10.1016/0926-6410(96)00032-8 8883926

[B5] Diéguez-Risco T. Aguado L. Albert J. Hinojosa J. A. (2015). Judging emotional congruency: explicit attention to situational context modulates processing of facial expressions of emotion. Biol. Psychol. 112, 27–38. 10.1016/j.biopsycho.2015.09.012 26450006

[B6] Dozolme D. Brunet-Gouet E. Passerieux C. Amorim M. A. (2015). Neuroelectric correlates of pragmatic emotional incongruence processing: empathy matters. PLoS ONE 10:e0129770. 10.1371/journal.pone.0129770 26067672 PMC4465748

[B7] Dunning J. P. Parvaz M. A. Hajcak G. Maloney T. Alia-Klein N. Woicik P. A.. (2011). Motivated attention to cocaine and emotional cues in abstinent and current cocaine users–an ERP study. Eur. J. Neurosci. 33, 1716–1723. 10.1111/j.1460-9568.2011.07663.x 21450043 PMC3086977

[B8] Egidi G. Nusbaum H. C. (2012). Emotional language processing: how mood affects integration processes during discourse comprehension. Brain Lang. 122, 199–210. 10.1016/j.bandl.2011.12.008 22325258

[B9] Flaisch T. Junghöfer M. Bradley M. M. Schupp H. T. Lang P. J. (2008a). Rapid picture processing: affective primes and targets. Psychophysiology 45, 1–10. 10.1111/j.1469-8986.2007.00600.x 17910736

[B10] Flaisch T. Stockburger J. Schupp H. T. (2008b). Affective prime and target picture processing: an ERP analysis of early and late interference effects. Brain Topogr. 20, 183–191. 10.1007/s10548-008-0045-6 18335309

[B11] Forgas J. P. (2001). Feeling and Thinking: The Role of Affect in Social Cognition. Cambridge, UK: Cambridge University Press.

[B12] Franken I. H. Muris P. Nijs I. van Strien J. W. (2008). Processing of pleasant information can be as fast and strong as unpleasant information: Implications for the negativity bias. Neth. J. Psychol. 64, 168–176. 10.1007/BF03076419

[B13] Gross J. J. (2002). Emotion regulation: affective, cognitive, and social consequences. Psychophysiology 39, 281–291. 10.1017/S0048577201393198 12212647

[B14] Hagoort P. Hald L. Bastiaansen M. Petersson K. M. (2004). Integration of word meaning and world knowledge in language comprehension. Science 304, 438–441. 10.1126/science.1095455 15031438

[B15] Herbert C. Junghofer M. Kissler J. (2008). Event related potentials to emotional adjectives during reading. Psychophysiology 45, 487–498. 10.1111/j.1469-8986.2007.00638.x 18221445

[B16] Hietanen J. K. Astikainen P. (2013). N170 response to facial expressions is modulated by the affective congruency between the emotional expression and preceding affective picture. Biol. Psychol. 92, 114–124. 10.1016/j.biopsycho.2012.10.005 23131616

[B17] Junghöfer M. Bradley M. M. Elbert T. R. Lang P. J. (2001). Fleeting images: a new look at early emotion discrimination. Psychophysiology 38, 175–178. 10.1111/1469-8986.3820175 11347862

[B18] Kensinger E. A. (2009). Remembering the details: Effects of emotion. Emot. Rev. 1, 99–113. 10.1177/1754073908100432 19421427 PMC2676782

[B19] Kissler J. Herbert C. Winkler I. Junghofer M. (2009). Emotion and attention in visual word processing—An ERP study. Biol. Psychol. 80, 75–83. 10.1016/j.biopsycho.2008.03.004 18439739

[B20] Koelsch S. Kasper E. Sammler D. Schulze K. Gunter T. Friederici A. D. (2004). Music, language and meaning: brain signatures of semantic processing. Nat. Neurosci. 7, 302–307. 10.1038/nn1197 14983184

[B21] Kutas M. Federmeier K. D. (2011). Thirty years and counting: finding meaning in the N400 component of the event-related brain potential (ERP). Annu. Rev. Psychol. 62, 621–647. 10.1146/annurev.psych.093008.131123 20809790 PMC4052444

[B22] Lang P. J. Bradley M. M. Cuthbert B. N. (1997). Motivated attention: affect, activation, and action, in Attention and Orienting: Sensory and Motivational Processes, Vol. 97, eds Lang P. J. Simons R. F. Balaban M. (New York, NY: Routledge), 135.

[B23] Low A. Lang P. J. Bradley M. M. (2005). What pops out during rapid picture presentation? Psychophysiology 42:S81 10.1111/j.1469-8986.2005.00342.x

[B24] McHugo M. Olatunji B. O. Zald D. H. (2013). The emotional attentional blink: what we know so far. Front. Hum. Neurosci. 7:151. 10.3389/fnhum.2013.00151 23630482 PMC3632779

[B25] Most S. B. Smith S. D. Cooter A. B. Levy B. N. Zald D. H. (2007). The naked truth: positive, arousing distractors impair rapid target perception. Cogn. Emot. 21, 964–981. 10.1080/02699930600959340

[B26] Nigam A. Hoffman J. E. Simons R. F. (1992). N400 to semantically anomalous pictures and words. J. Cogn. Neurosci. 4, 15–22. 10.1162/jocn.1992.4.1.15 23967854

[B27] Ohman A. Flykt A. Esteves F. (2001). Emotion drives attention: detecting the snake in the grass. J. Exp. Psychol. 130:466. 10.1037/0096-3445.130.3.466 11561921

[B28] Öhman A. Hamm A. Hugdahl K. (2000). Cognition and the autonomic nervous system: Orienting, anticipation, and conditioning, in Handbook of Psychophysiology 2nd edn. eds Cacioppo J. T. Tassinary L. G. Berntson G. G. (New York, NJ: Cambridge University Press) 533–575.

[B29] Otten M. Van Berkum J. J. (2007). What makes a discourse constraining? Comparing the effects of discourse message and scenario fit on the discourse-dependent N400 effect. Brain Res. 1153, 166–177. 10.1016/j.brainres.2007.03.058 17466281

[B30] Pessoa L. (2008). On the relationship between emotion and cognition. Nat. Rev. Neurosci. 9, 148. 10.1038/nrn2317 18209732

[B31] Potter M. C. Staub A. O'Connor D. H. (2002). The time course of competition for attention: attention is initially labile. J. Exp. Psychol. Hum. Percept. Perform. 28:1149. 10.1037/0096-1523.28.5.1149 12421061

[B32] Schupp H. T. Flaisch T. Stockburger J. Junghöfer M. (2006a). Emotion and attention: event-related brain potential studies. Prog. Brain Res. 156, 31–51. 10.1016/S0079-6123(06)56002-9 17015073

[B33] Schupp H. T. Junghöfer M. Weike A. I. Hamm A. O. (2003). Attention and emotion: an ERP analysis of facilitated emotional stimulus processing. Neuroreport 14, 1107–1110. 10.1097/01.wnr.0000075416.59944.49 12821791

[B34] Schupp H. T. Junghöfer M. Weike A. I. Hamm A. O. (2004a). The selective processing of briefly presented affective pictures: an ERP analysis. Psychophysiology 41, 441–449. 10.1111/j.1469-8986.2004.00174.x 15102130

[B35] Schupp H. T. Ohman A. Junghöfer M. Weike A. I. Stockburger J. Hamm A. O. (2004b). The facilitated processing of threatening faces: an ERP analysis. Emotion 4:189. 10.1037/1528-3542.4.2.189 15222855

[B36] Schupp H. T. Stockburger J. Bublatzky F. Junghöfer M. Weike A. I. Hamm A. O. (2007a). Explicit attention interferes with selective emotion processing in human extrastriate cortex. BMC Neurosci. 8:16. 10.1186/1471-2202-8-16 17316444 PMC1808466

[B37] Schupp H. T. Stockburger J. Codispoti M. Junghöfer M. Weike A. I. Hamm A. O. (2006b). Stimulus novelty and emotion perception: the near absence of habituation in the visual cortex. Neuroreport 17, 365–369. 10.1097/01.wnr.0000203355.88061.c6 16514360

[B38] Schupp H. T. Stockburger J. Codispoti M. Junghöfer M. Weike A. I. Hamm A. O. (2007b). Selective visual attention to emotion. J. Neurosci. 27, 1082–1089. 10.1523/JNEUROSCI.3223-06.2007 17267562 PMC6673176

[B39] Shapiro K. L. Raymond J. E. Arnell K. M. (1997). The attentional blink. Trends Cogn. Sci. 1, 291–296. 10.1016/S1364-6613(97)01094-2 21223931

[B40] Steinhauer K. Alter K. Friederici A. D. (1999). Brain potentials indicate immediate use of prosodic cues in natural speech processing. Nat. Neurosci. 2, 191. 10.1038/5757 10195205

[B41] Tipples J. Sharma D. (2000). Orienting to exogenous cues and attentional bias to affective pictures reflect separate processes. Br. J. Psychol. 91, 87–97. 10.1348/000712600161691 10717773

[B42] Vinck M. Bosman C. A. (2016). More gamma more predictions: gamma-synchronization as a key mechanism for efficient integration of classical receptive field inputs with surround predictions. Front. Syst. Neurosci. 10:35. 10.3389/fnsys.2016.00035 27199684 PMC4842768

[B43] Vuilleumier P. (2005). How brains beware: neural mechanisms of emotional attention. Trends Cogn. Sci. 9, 585–594. 10.1016/j.tics.2005.10.011 16289871

[B44] Vuilleumier P. Richardson M. P. Armony J. L. Driver J. Dolan R. J. (2004). Distant influences of amygdala lesion on visual cortical activation during emotional face processing. Nat. Neurosci. 7, 1271. 10.1038/nn1341 15494727

[B45] Wang L. Bastiaansen M. Yang Y. Hagoort P. (2011). The influence of information structure on the depth of semantic processing: How focus and pitch accent determine the size of the N400 effect. Neuropsychologia 49, 813–820. 10.1016/j.neuropsychologia.2010.12.035 21195102

[B46] Weinberg A. Ferri J. Hajcak G. (2013). Interactions between attention and emotion. Insights from the late positive potential, in Handbook of Cognition and Emotion, eds Robinson M. D. Watkins E. R. Harmon-Jones E. (New York, NY: Guilford Press), 35–54.

[B47] Yiend J. (2010). The effects of emotion on attention: a review of attentional processing of emotional information. Cogn. Emot. 24, 3–47. 10.1080/02699930903205698

